# Reduced Nephrin Tyrosine Phosphorylation Enhances Insulin Secretion and Increases Glucose Tolerance With Age

**DOI:** 10.1210/endocr/bqae078

**Published:** 2024-07-02

**Authors:** Casey R Williamson, Nina Jones

**Affiliations:** Department of Molecular and Cellular Biology, University of Guelph, Guelph, ON, N1G 2W1, Canada; Department of Molecular and Cellular Biology, University of Guelph, Guelph, ON, N1G 2W1, Canada

**Keywords:** nephrin, β cell, islet, tyrosine phosphorylation, glucose tolerance, diabetes

## Abstract

**Background:**

Nephrin is a transmembrane protein with well-established signaling roles in kidney podocytes, and a smaller set of secretory functions in pancreatic β cells are implicated in diabetes. Nephrin signaling is mediated in part through its 3 cytoplasmic YDxV motifs, which can be tyrosine phosphorylated by high glucose and β cell injuries. Although in vitro studies demonstrate these phosphorylated motifs can regulate β cell vesicle trafficking and insulin release, in vivo evidence of their role in this cell type remains to be determined.

**Methods:**

To further explore the role of nephrin YDxV phosphorylation in β cells, we used a mouse line with tyrosine to phenylalanine substitutions at each YDxV motif (nephrin-Y3F) to inhibit phosphorylation. We assessed islet function via primary islet glucose-stimulated insulin secretion assays and oral glucose tolerance tests.

**Results:**

Nephrin-Y3F mice successfully developed pancreatic endocrine and exocrine tissues with minimal structural differences. Unexpectedly, male and female nephrin-Y3F mice showed elevated insulin secretion, with a stronger increase observed in male mice. At 8 months of age, no differences in glucose tolerance were observed between wild-type (WT) and nephrin-Y3F mice. However, aged nephrin-Y3F mice (16 months of age) demonstrated more rapid glucose clearance compared to WT controls.

**Conclusion:**

Taken together, loss of nephrin YDxV phosphorylation does not alter baseline islet function. Instead, our data suggest a mechanism linking impaired nephrin YDxV phosphorylation to improved islet secretory ability with age. Targeting nephrin phosphorylation could provide novel therapeutic opportunities to improve β cell function.

Nephrin is an essential membrane protein with expression mostly restricted to kidney podocytes and pancreatic β cells (1, 2). In podocytes, nephrin is a core component of the slit diaphragm, a filtration barrier that prevents large serum proteins from entering the urine. The extracellular region of nephrin physically forms part of the slit diaphragm architecture, and its cytoplasmic region operates as an important signaling hub that binds myriad interactors (3). Notably, nephrin's cytoplasmic region can be phosphorylated at 3 YDxV motifs (corresponding to Y1176, Y1193, and Y1217 in humans). These residues recruit binding partners such as podocin, PLCγ, and Nck, allowing for MAPK, calcium, and actin regulation, respectively (4-6). The Nck family of adaptor proteins are especially important, as the multivalency of their 3 SH3 domains, along with nephrin's 3 YDxV motifs, catalyzes phase separation of actin regulatory centers (7). Importantly, altered phosphorylation of YDxV motifs is observed in a variety of kidney pathologies (8-12). Moreover, mice with tyrosine to phenylalanine substitutions at these 3 motifs (nephrin-Y3F) develop progressive kidney disease and show susceptibility to podocyte injury (8). Independent of phosphorylation, nephrin can also regulate vesicle trafficking, cell polarity, and cell survival/apoptosis through interactions with VAMP2, aPKC, CD2AP, and Akt (13-19). To date, however, most of these functions have been established in the context of podocytes, and nephrin's role in the pancreas remains largely unexplored.

In the pancreas, nephrin is found at the β cell plasma membrane and in insulin-containing vesicles (20). Despite this exclusive localization, nephrin's expression in the pancreas has shown several inconsistencies, including instances where no nephrin is detected across the pancreas (20, 21) or expression appears in glandular cells (22). Nevertheless, elevated nephrin expression in β cells is associated with enhanced glucose-stimulated insulin secretion (GSIS), increased insulin content, and a greater number of secretory granules (20). High glucose also stimulates YDxV phosphorylation at tyrosines Y1176 and/or Y1193, inducing nephrin endocytosis in a process dependent on dynamin and actin remodeling (20, 23). Furthermore, nephrin expression in β cells modulates insulin receptor-mediated cell survival and growth signaling, with nephrin overexpression leading to activation of PI3K, Akt, mTORC1, and p70S6K, though these responses appear independent of YDxV phosphorylation (24). Ultimately, nephrin's role in β cells appears to be clinically relevant, as nephrin expression is reduced in islets from individuals with type 2 diabetes, and patients with nephrin mutations show glucose intolerance (20, 24). Together these studies reveal the importance of nephrin signaling outside the kidney, yet the in vivo requirement for nephrin YDxV phosphorylation in an alternate setting has yet to be determined.

In this study, we sought to investigate pancreatic structure and islet function in our previously validated nephrin-Y3F mice (8). First, we interrogated new datasets that highlighted nephrin's expression in multiple pancreatic secretory cell types. Next, we assessed pancreatic structure in nephrin-Y3F mice, which had minimal alterations in exocrine structures and islet morphology. In line with this finding, loss of nephrin YDxV phosphorylation did not impair GSIS but instead led to enhanced insulin secretion and glucose tolerance with age. Altogether, signaling through these tyrosines modulates β cell activity in a manner that might be more complex than previously understood, and further research exploring nephrin as a therapeutic target to promote insulin secretion is therefore warranted.

## Materials and Methods

### Animal Care and Study Approval

Animal housing protocols were maintained in accordance with the guidelines set by the Canadian Council of Animal Care. Approval for all experimental procedures involving mice was provided by the Animal Care Committee at the University of Guelph (AUP 4181). All mice were housed with a 12-hour light/12-hour dark cycle and were given free access to 14% fat chow (Teklad 2914, Inotiv) and drinking water.

### Generation of Genetically Modified Mouse Lines

Mice with homozygous *Nphs1*^Y3F/Y3F^ alleles (nephrin-Y3F mice; MGI: 7624348) were previously generated and validated (8). Nephrin-Y3F mice carried 3 Y→F missense mutations (p.Y1191F, p.Y1208F, and p.Y1232F), which correspond to the human tyrosine residues Y1176, Y1193, and Y1217. To refresh the colony genetic backgrounds, nephrin-Y3F mice were bred and maintained on the C57BL/6N background using wild-type (WT) C57BL/6NCrl-Elite-specific and opportunistic pathogen free mice (Charles River; RRID: IMSR_CRL:475) every 5 to 10 generations. Mice were genotyped before experimentation by polymerase chain reaction and resolved on 2% agarose gels (8). Mice from a minimum of 2 litters for each genotype per experiment were used. Mature adult mice (4-12 months of age) were used in all instances, unless otherwise noted, and mice were age-matched for all statistical comparisons.

### Islet Isolation

For pancreatic dissection, the initial portion of the duodenum and the colon were collectively clamped with a hemostat, the intestinal tract was cut, and all attached organs were placed in cold Hank's balanced salt solution (HBSS; 311-513-CL; Wisent Bioproducts) with 5 mM sodium bicarbonate, 1.25 mM calcium chloride, and 1 mM magnesium chloride. Pancreata were torn from intestinal tracts using forceps, minced into approximately 0.1 mm^3^ pieces, and placed in 2 mL of a 1.081 Wuensch U/mL solution of Liberase TL (05401020001; Roche) in HBSS (25). Pancreata were digested by incubating in a 37 °C water bath for 20 minutes, with consistent mixing by inversion every 3 minutes. Following incubation, samples were forcefully shaken for 30 seconds, and 8 mL of cold HBSS with 10% fetal bovine serum (098150; Wisent Bioproducts) was added to the digested pancreas mixture to stop digestion. Samples were centrifuged at 500×g for 1 minute at room temperature, supernatants were discarded, and pellets were resuspended in 5 mL cold HBSS. Twenty islets with approximately mid-range diameters were then handpicked using a Leica MZ125 dissection microscope and a p1000 pipette tip to be placed in a clean culture dish with cold HBSS. The selected islets were handpicked a second time to further purify islets from pancreatic acinar tissue. Islet identity was confirmed by staining additional fractions of isolated islets with 1:5 dithizone (DTZ; D5130; Sigma-Aldrich) in Dulbecco's PBS (211-410-XK; Wisent Bioproducts) for 1 minute at room temperature.

### Glucose-Stimulated Insulin Secretion Assay

Ten islets of equivalent size were placed in 12-well plate wells containing 2 mL 0.2 μm-filter-sterilized Krebs-Ringer Buffer (311-605-CL; Wisent Bioproducts) with 10 mM HEPES (4-(2-hydroxyethyl)-1-piperazineethanesulfonic acid), 24 mM sodium bicarbonate, 2.5 mM calcium chloride, 1 mM magnesium chloride, 0.1% bovine serum albumin (A9418; Sigma-Aldrich), pH 7.4, and either 6.5 mM or 22 mM D-glucose (G8769; Sigma-Aldrich). Islets were incubated at 37 °C and 5% CO_2_ for 2 hours before 1 mL culture media was collected, snap-frozen on dry ice, and stored at −80 °C. Insulin concentration in culture media was assessed using the mouse insulin ELISA kit from Crystal Chem (90080, RRID: AB_2783626), as per manufacturer's instructions.

### Oral Glucose Tolerance Tests

Mice were fasted for 6 hours prior to oral glucose tolerance tests (OGTTs). Fasting cages included only water, fresh bedding, and nondigestible enrichment materials. After fasting, mice were weighed, blood glucose (BG) was sampled by 1 mm tail snips, and BG was measured using OneTouch Verio test strips and glucometer (LifeScan). Immediately following BG measurement, mice were administered 2 mg/g bodyweight of D-glucose (G8769; Sigma-Aldrich; 0.2 μm-filter-sterilized 250 mg/mL in diH_2_O) by gavage using a ball-tipped needle. All volumes administered were less than 1% body weight. Tail clots were gently removed with sterile scissors, and BG was successively measured at the indicated time points.

### Brightfield and Immunofluorescence Microscopy

For brightfield microscopy, pancreatic gastric lobes were fixed in 10% buffered formalin (Thermo Fisher Scientific) for a minimum of 48 hours and submitted to Animal Health Laboratories—Histotechnology (University of Guelph) for processing. Tissues were paraffin-embedded and 4 4 µm sections, spaced out by 300 µm, were stained using hematoxylin and eosin (H&E). Three mice per group were used to visualize 17 to 39 islets per mouse and viewed using a Leica DM1000 and ToupView software (ToupTek). Islet area and internuclear spacing were quantified using ImageJ software. Distance between nuclear clusters was performed by placing ellipses at each large space between nuclei, without overlap with previous ellipse areas, and measuring the minimal Feret's diameter. Only the 3 largest diameters per islet were quantified (or 1-2 measurements when diameters were <500 µm^2^).

For immunofluorescence imaging, pancreatic tissue from the upper portion of the duodenal lobe was placed in Shandon Cryomatrix (Richard-Allan Scientific), snap-frozen on dry ice, and stored at −80 °C. Two 8 μm sections per mouse, spaced out by 300 µm, were fixed in cold acetone for 10 minutes, washed with PBS (137 mM NaCl, 2.7 mM KCl, 10 mM Na2HPO4, and 1.8 mM KH2PO4; pH 7.4), and blocked with 10% normal goat serum (NGS; Santa Cruz Biotechnology) in PBS for 1 hour at room temperature. Sections were then incubated with rabbit anti-insulin (H-86; sc-9168; 1:200; Santa-Cruz Biotechnology; RRID: AB_2126540) in 10% NGS for 1 hour at room temperature, washed 3 times with PBS, and incubated with donkey anti-rabbit (Alexa Fluor 647; A31573; 1:100; Thermo Fisher Scientific; RRID: AB_2536183) in 10% NGS while light-protected for 45 minutes at room temperature. After another 3 PBS washes, samples were light-protected and incubated with mouse anti-Glucagon (Alexa Fluor 488-conjugated; ICACLS; 53-9743-82; 1:100; Thermo Fisher Scientific; RRID: AB_2574455) for 1 hour at room temperature, followed by 3 PBS washes. Sections were stained with Hoechst 33258 (H1398; 1:2000; Invitrogen) for 2 minutes at room temperature to stain nuclei and mounted using ProLong Diamond Antifade Mountant (Invitrogen). For each mouse, 16 to 28 islets were visualized on an Echo Revolve (Model RVL2-K2), brightness was adjusted to a negative control pancreatic serial section without target antibody, and ImageJ software was used to quantify the visualized area.

### Data Mining and Repositories

System-wide tissue localization of *NPHS1* was accessed using the Human Protein Atlas (HPA), where the consensus dataset of HPA and Genotype-Tissue Expression (GTEx) was used for RNA expression (available at https://www.proteinatlas.org/ENSG00000161270-NPHS1/tissue; accessed July 2023) (26). Single-cell RNA sequencing data of *NPHS1* expression in the pancreas was accessed using the HPA single cell atlas (https://www.proteinatlas.org/ENSG00000161270-NPHS1/single+cell+type/pancreas; accessed July 2023) (27) and the University of California Santa Cruz Single Cell Browser using the dataset provided by Enge et al (https://cells.ucsc.edu/?ds=adultPancreas&gene=NPHS1) (28, 29).

### Statistics and Data Visualization

Statistical analyses were performed using GraphPad Prism software (version 10.0.2). Comparisons between 2 unpaired groups with normal distribution were performed using 2-tailed Student's *t*-test. Statistically significant differences in insulin secretion between glucose treatments, genotypes, and sexes were assessed using a 3-way ANOVA. Simple main effects and all other statistical analyses were performed using a 2-way ANOVA with a post hoc Tukey's honest significant difference test where applicable. Significance was established using an α level of .05, and visualization of significant differences for separate factors was distinguished using asterisks (*) or obelisks (†).

## Results

### Nephrin is Detected in Endocrine and Exocrine Pancreatic Cell Types

**Figure 1.**
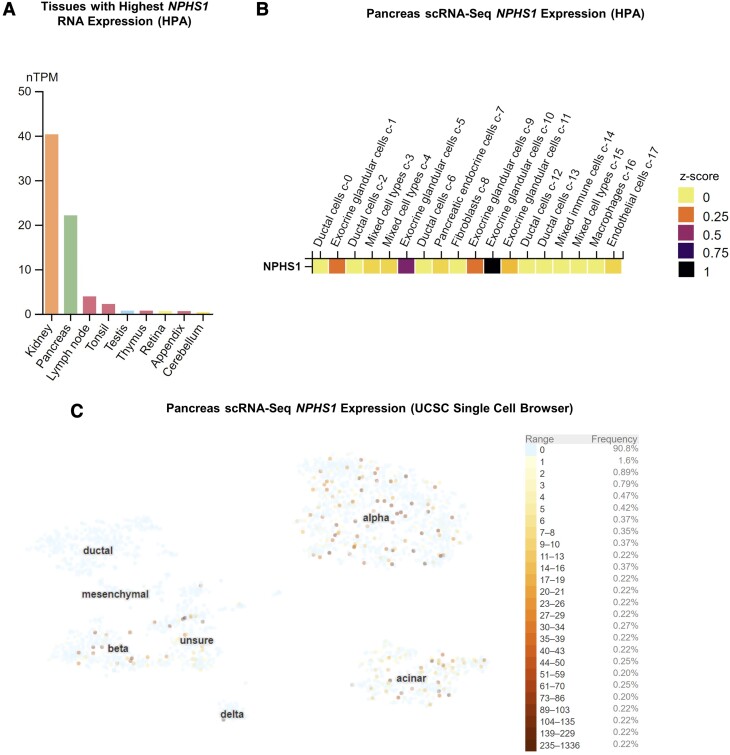
Nephrin (*NPHS1*) mRNA expression profile showing enrichment in pancreas and pancreatic exocrine and endocrine cell types. (A) HPA and gTEX portal datasets were arranged to show the top 10 tissues for *NPHS1* mRNA expression in humans (26). (B, C) scRNA-Seq datasets from the HPA (27) (B) and University of California Santa Cruz Single Cell Browser (29) (C) show that *NPHS1* mRNA is expressed in a subset of endocrine islet cells (cluster 7 from HPA, mostly consisting of α and β cells) and exocrine acinar cells (clusters 1, 5, 9, 10, and 11 from HPA) but is absent in exocrine ductal cells (clusters 2, 6, 12, and 13 from HPA). Abbreviations: HPA, Human Protein Atlas; scRNA-Seq, single-cell RNA sequencing.

Despite multiple roles of nephrin being reported in β cells, its detection and localization in the pancreas have shown inconsistent results (2, 20-22), potentially due to challenges with antibody specificity. As minimal studies have examined pancreatic *NPHS1* mRNA expression (20), we first assessed nephrin's mRNA distribution among updated expression databases including the HPA, the GTEx Portal, and the University of California Santa Cruz Single Cell Browser. Combined datasets from the HPA and GTEx confirmed pancreatic nephrin mRNA expression, with the pancreas being the only major source of nephrin in tissues outside the kidney (Fig. 1A) (26). Single-cell RNA sequencing datasets also confirmed nephrin expression in β cells but revealed an unexpectedly greater degree of nephrin expression in exocrine and other endocrine cell types, notably acinar and α cells, respectively (Fig. 1B and 1C) (27, 29).

### Nephrin-Y3F Mice Develop Islets With Minor Structural Aberrations and an Enhanced Insulin Secretion Ability

**Figure 2.**
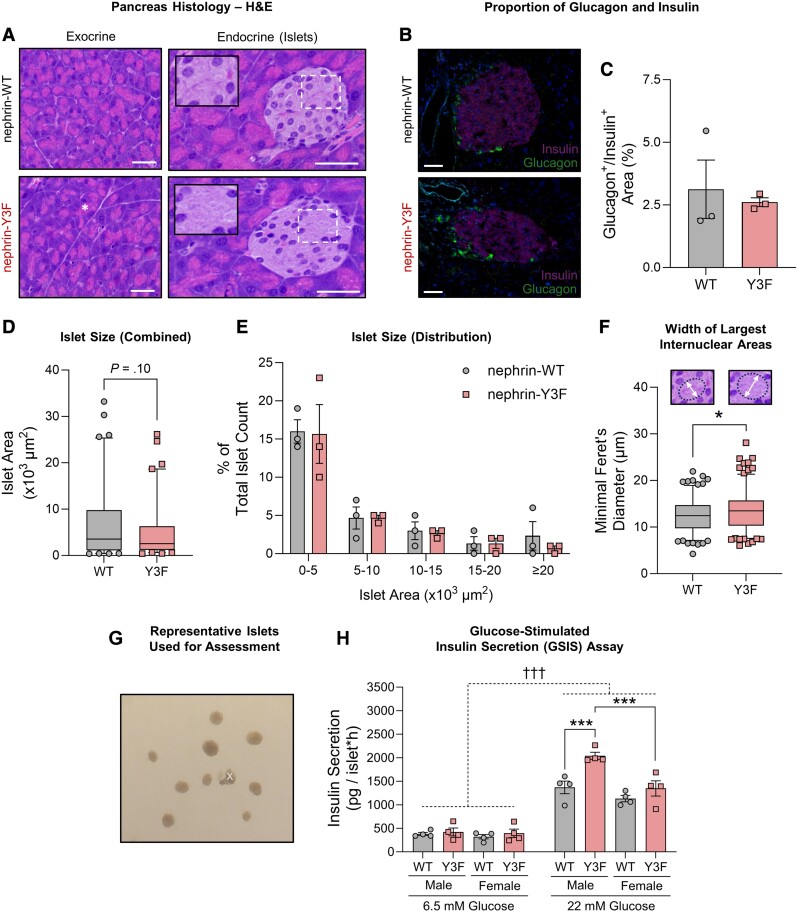
Nephrin-Y3F mice show minor alterations in islet structure and have a moderate enhancement of glucose-dependent insulin secretion. (A) Brightfield images of H&E-stained pancreatic exocrine and endocrine (islet) tissues from adult male WT or nephrin-Y3F mice. Nephrin-Y3F mice developed structurally comparable zymogen granules (asterisk) and islets but had distinct clustering of islet nuclei and larger gaps observed between these clusters (insets). (B) Immunofluorescence images showing similar insulin and glucagon staining between adult male WT and nephrin-Y3F mice. (C) Percentage quantification of glucagon-stained area to insulin-positive area from (B) (16-28 islets per mouse; 3 mice per group). (D) Box-and-whisker plot showing all islet area quantifications per genotype from images in (A) (17-39 islets per mouse; 3 mice per group), with no significant differences between genotypes. (E) Percentage distribution of islets from (A) within specified size ranges (3 mice per group). (F) Width measurements of spaces between clustered nuclei. Nephrin-Y3F mice had larger spaces between nuclear clusters, as measured using the minimal Feret's diameter of an ellipse between nuclei (examples shown in graph), with the 3 largest ellipses being quantified per islet (17-39 islets per mouse; 3 mice per group). (G) Representative image of islets to be used for GSIS assay. Islets with attached acinar tissue (noted by “x”) were omitted from assessment. (H) GSIS assays using primary islets show sex-specific enhancement of insulin release in nephrin-Y3F mice (n = 4). Bars show mean ± SEM. Box-and-whisker plots show median ± 5-95th percentiles. Statistical significance between 2 groups was determined using a Student's *t*-test, and a 3-way ANOVA with a post hoc Tukey's test was used for the GSIS assay. Significant differences are indicated as * for *P* < .05 and †††/*** for *P* < .001. Scale bars are 40 µm. Abbreviations: GSIS, glucose-stimulated insulin secretion; H&E, hematoxylin and eosin; WT, wild-type

To investigate the role of nephrin YDxV signaling in the development of exocrine and/or endocrine tissues, we visualized H&E-stained pancreata in WT and nephrin-Y3F knockin mice and compared their structure to WT controls. Exocrine tissue in nephrin-Y3F mice showed no overt structural changes, with similar acinar cell shape, orientation, and proportion of zymogen granules (Fig. 2A). Consistent with previous observations in mice with β cell-specific nephrin deletion (24), nephrin-Y3F mice developed islets with no discernable signs of cell stress, such as pyknotic nuclei, immune infiltration, or dysplasia (Fig. 2A). Furthermore, we did not detect differences in the proportion of α to β cells, as measured by glucagon and insulin immunofluorescence staining (Fig. 2B and 2C). To detect potential differences in islet size and/or islet neogenesis, we quantified islet area and size distribution. While nephrin-Y3F islets were slightly smaller than WT mice, this difference was not significant (*t* = 1.659; *df* = 160; *P* = .099) (Fig. 2D and 2E), and there were also no significant differences in the percentage of endocrine to exocrine area (0.260 ± 0.107% vs 0.257 ± 0.056%) (data not shown). In contrast, a distinct difference in nephrin-Y3F islet nuclear arrangement was observed, with these islets having greater clustering of nuclei, with larger distances between these clusters that appear to be found near the β cell apical and islet stromal regions (inset in Fig. 2A). To quantify these enlarged spaces, ellipses were drawn between nuclei at the 3 largest spaces per islet and the widths of the ellipses were quantified. Indeed, nephrin-Y3F mice had significantly larger widths at these pronounced spaces (*t* = 2.332; *df* = 360; *P* = .020) (Fig. 2F).

Next, to determine if any of these structural alterations might correlate with differences in insulin release, we isolated islets from WT or nephrin-Y3F mice (representative images of isolated islets in Fig. 2G) and measured GSIS. Unexpectedly, we found an enhancement of islet secretion ability in nephrin-Y3F mice [simple main genotype effect at 22 mM glucose; *F*(1,12) = 14.50; *P* = .0025], and the degree of secreted insulin was greater in nephrin-Y3F males than nephrin-Y3F females (Fig. 2H, post hoc Tukey's honest significant difference between nephrin-Y3F male and nephrin-Y3F female islets at 22 mM glucose; *P* = .0003). Together, these results support previous findings that nephrin YDxV phosphorylation affects islet function and insulin release, although the loss of this signaling axis leads to an unexpected enhancement of insulin secretion, rather than impairment.

### Adult Nephrin-Y3F Mice Show no Alterations in Glucose Tolerance

**Figure 3.**
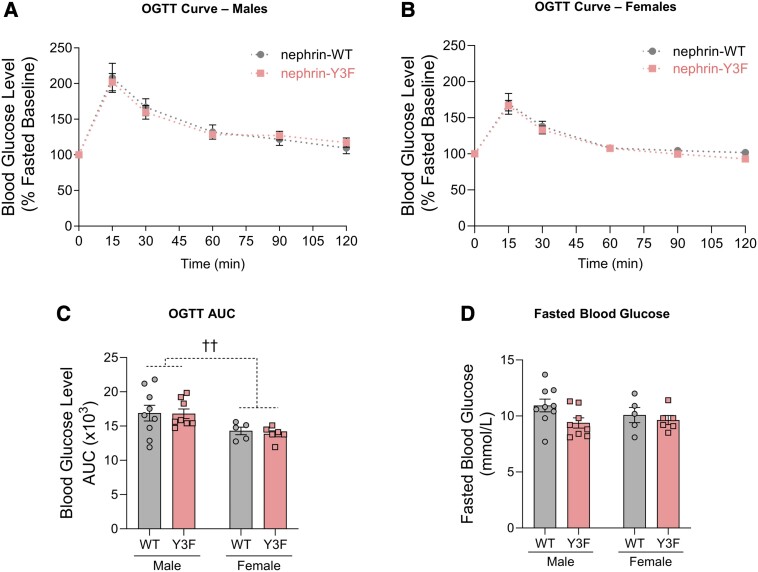
Adult nephrin-Y3F mice show normal glucose tolerance. (A-B) OGTTs were performed using 2 mg of D-glucose per gram of bodyweight on 6-hour-fasted male (A; n = 8-9) or female (B; n = 5-6) WT or nephrin-Y3F mice (8 months of age). (C) AUC measurements for the respective OGTT curves in (A) and (B). (D) 6-hour fasting blood glucose concentrations on WT or nephrin-Y3F mice (n = 5-9) (8 months of age). Bars show mean ± SEM. Statistical significance was determined using 2-way ANOVA with a post hoc Tukey's test. Significant main effects differences are indicated as †† for *P* < .01. Abbreviations: AUC, area under the curve; OGTT, oral glucose tolerance test; WT, wild-type.

To determine if these islet structural and functional differences affect glucose clearance at the systemic level, we performed OGTTs on WT and nephrin-Y3F mice. Due to nephrin's detection in exocrine tissues, oral administration of glucose, rather than intraperitoneal, was performed to account for potential alterations in exocrine/endocrine crosstalk. Surprisingly, we found no evidence of altered blood glucose tolerance in both male and female mice at 4 months of age (Supplementary Fig. S1A-S1C) (30) and 8 months of age (Fig. 3A-C). Area under the curve analysis showed generally higher glucose tolerance in 8-month-old female mice compared with male mice (Fig. 3C), consistent with previous reports (31). We also observed equivalent 6-hour-fasted blood glucose levels between all sexes and genotypes (Supplementary Fig. S1D and S3D) (30). However, reflecting the increased insulin response observed in male nephrin-Y3F islets, we did see slightly lower fasted blood glucose in 8-month-old male mice (Fig. 3D), but this difference was not significant (*P* = .139). Ultimately, the loss of nephrin YDxV signaling does not impair islet function, and the enhanced insulin secretion phenotype we observed in nephrin-Y3F islets ex vivo does not impact blood glucose homeostasis in baseline health conditions.

### Aged Nephrin-Y3F Mice Display Increased Glucose Tolerance

**Figure 4.**
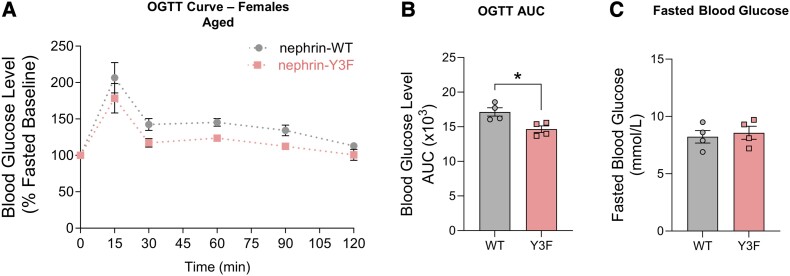
Improved glucose tolerance is observed in aged nephrin-Y3F mice. (A) OGTTs were performed at 16 months of age using 2 mg of D-glucose per gram of bodyweight on 6-hour-fasted female WT or nephrin-Y3F mice (n = 4). (B) AUC measurements for the respective OGTT curves in (A). (C) 6-hour fasting blood glucose concentrations on 16-month-old female WT or nephrin-Y3F mice (n = 4). Bars show mean ± SEM. Statistical significance was determined using Student's *t*-test. Significant difference is indicated as * for *P* < .05. Abbreviations: AUC, area under the curve; OGTT, oral glucose tolerance test; WT, wild-type.

To help explain the discrepancy between our enhanced GSIS and unchanged OGTTs, it is important to note that our GSIS assessments were observed at a later age (12 months of age), which might have influenced the secretory phenotype. Furthermore, our previous investigations of nephrin-Y3F mice showed kidney phenotypes that increase with age (8). Therefore, to determine if aged nephrin-Y3F mice have differences in islet function, we performed an additional OGTT evaluation at 16 months of age. Consistent with the increased insulin release observed ex vivo (Fig. 2B), aged nephrin-Y3F mice showed greater glucose tolerance (area under the curve Student's *t*-test; *t* = 3.216, *df* = 6; *P* = .0182), with no differences in fasted blood glucose (Fig. 4A-C). Knowing nephrin-Y3F islets had structural differences at adult ages that might vary over time, we also visualized islets in aged nephrin-Y3F mice and found they maintained a similar clustered nuclear arrangement and structural characteristics as those observed at younger ages (Supplementary Fig. S2) (30). Together, nephrin-Y3F mice have increased glucose tolerance at later ages, and this difference is concomitant with a similar islet morphology that was observed at younger ages.

## Discussion

Nephrin maintains specialized functions in podocytes and β cells using phospho-dependent and phospho-independent mechanisms. Just as nephrin-Y3F mice can develop sufficient podocyte structure in the absence of injury/aging, we now show that nephrin YDxV phosphorylation is not required for baseline islet development and function in vivo. However, loss of this signaling axis enhances insulin secretion ex vivo and in aged mice, suggesting that these residues are potentially involved in context-dependent β cell functions. Such contexts might relate to cell survival, senescence, biomechanical stimuli, and stress responses, as these processes can be altered with age and from many islet pathologies (32, 33).

Outside of these contexts, the normal β cell insulin secretion we observed in nephrin-Y3F mice (Fig. 3 and Supplementary Fig. S1) (30) provides an important clue for defining nephrin's main role in islets. Independently of YDxV phosphorylation, nephrin is capable of binding interactors involved in actin regulation, cell polarity, cell-cell junctions, membrane potential regulation, survival/anti-apoptotic signaling, and vesicle trafficking (15, 16, 19, 34-36). Previously, a conditional β cell-specific nephrin knockout showed that nephrin is required for proper glucose handling (24); therefore, our findings suggest that phospho-YDxV-independent functions are sufficient to provide the bulk of nephrin's essential role in insulin secretion. This notion is contrary to the available evidence that suggests phosphorylation at these residues is important for insulin vesicle endocytosis and GSIS (20, 23). Yet, it remains possible YDxV phosphorylation plays crucial roles in pathological responses where rapid actin reorganization and cell survival signals are needed. Such a role would reflect our observations in podocytes, where nephrin-Y3F mice are susceptible to acute and age-related injury (8). These large stress responses might require the multivalency of nephrin's 3 YDxV sites to undergo phase separation for efficient actin regulation (7). Nonetheless, our current evidence suggests this phase separation ability is not required for normal islet function.

Next, as previous studies have shown that nephrin-Y3F expression is associated with *decreased* insulin secretion, it remains an important question why we have observed an *increased* ability ex vivo and with age. As one potential explanation, nephrin phosphorylation at these 3 motifs is associated with actin polymerization (6, 37, 38), while the loss of this actin polymerization might increase GSIS by allowing easier transport of reserve vesicles to the plasma membrane (39, 40). As another explanation, previous studies have established nephrin YDxV's impact on GSIS using WT and nephrin-Y3F overexpression (23), and it has been observed that these nephrin-Y3F mutants have impaired endocytosis and trafficking (41). Thus, overexpression of these constructs might have led to abnormally high levels of nephrin protein that negatively alter GSIS through their impaired trafficking. In contrast, nephrin-Y3F mice retain an expression level representative of its regulation in vivo. If nephrin-Y3F in vivo leads to the increased nephrin surface levels we have previously observed (41), it is possible that this leads to greater survival signals through activation of Akt, mTORC1, and S6K via IR-B (24), and/or decreased NF-κB through its PDZ-binding motif (16). Increased survival signals might have been protective during the processes of primary islet isolation and/or aging, leading to the observed enhancement of GSIS in these contexts. Lastly, there is evidence that different YDxV motifs have opposing effects on GSIS, where overexpression of Y1217F mutants led to improved insulin secretion upon protamine sulfate injury (23). Absence of this phosphorylated residue in vivo might have had positive effects that outweigh loss of phosphorylation at other YDxV sites, making this specific tyrosine a potential clinical target for pathologies like type 2 diabetes.

## Limitations

This study is only a first step in exploring this unexpected role of nephrin, and future validation is needed. First, only nephrin-Y3F female mice were available to show improved glucose tolerance with age (Fig. 4), while we observed the insulin secretion phenotype in male mice (Fig. 2H). Thus, further investigation of islet function in both sexes at advanced ages would greatly support these findings. Additionally, our characterization of nephrin-Y3F islet structure (Fig. 2B-F) should be performed across the entire pancreas, as our quantified tissues were from the gastric and upper duodenal pancreatic lobes, and pancreatic islet distribution is highly variable across multiple lobes. With regard to these structural insights, the observed nuclear clusters and internuclear spaces in nephrin-Y3F islets (Fig. 2F) remain to be explained. This feature was not associated with overall islet function, as it was detected at ages with unchanged glucose tolerance, but this morphology might affect islet insulin secretion during various pathologies and should be investigated across multiple contexts. Interestingly, this increased spacing was found near apical β cell and stromal regions (Fig. 2A), so it would be helpful to assess the potential roles of nephrin in this area, such as in β cell primary cilia (42), or in other cell types like islet pericytes (43). Nephrin transcripts are indeed observed in other cell types, including exocrine acinar cells and alpha cells (Fig. 1B and 1C). Thus, future research should assess secretory functions from these other cell types as well, which might have indirectly affected our secretory phenotype in β cells. While acknowledging these valuable next steps, our central insights into the impacts of nephrin YDxV signaling in vivo remain important for our understanding of islet physiology. As studies on pancreatic nephrin have been limited in recent years, we hope these findings warrant future investigations into nephrin's spatiotemporal signaling in this important tissue setting.

## Data Availability

Some or all datasets generated during and/or analyzed during the current study are not publicly available but are available from the corresponding author on reasonable request.

## References

[1] Ruotsalainen V, Ljungberg P, Wartiovaara J, et al Nephrin is specifically located at the slit diaphragm of glomerular podocytes. Proc Natl Acad Sci U S A. 1999; 96(14):7962‐7967. 10393930 10.1073/pnas.96.14.7962 PMC22170

[2] Palmén T, Ahola H, Palgi J, et al Nephrin is expressed in the pancreatic beta cells. Diabetologia. 2001; 44(10):1274‐1280. 11692176 10.1007/s001250100641

[3] Martin CE, Jones N. Nephrin signaling in the podocyte: an updated view of signal regulation at the slit diaphragm and beyond. Front Endocrinol. 2018; 9:302. 10.3389/fendo.2018.00302 PMC5996060 29922234

[4] Huber TB, Köttgen M, Schilling B, Walz G, Benzing T. Interaction with podocin facilitates nephrin signaling. J Biol Chem. 2001; 276(45):41543‐41546. 11562357 10.1074/jbc.C100452200

[5] Harita Y, Kurihara H, Kosako H, et al Phosphorylation of nephrin triggers Ca2+ signaling by recruitment and activation of phospholipase C-γ1. J Biol Chem. 2009; 284(13):8951‐8962. 19179337 10.1074/jbc.M806851200 PMC2659252

[6] Jones N, Blasutig IM, Eremina V, et al Nck adaptor proteins link nephrin to the actin cytoskeleton of kidney podocytes. Nature. 2006; 440(7085):818‐823. 16525419 10.1038/nature04662

[7] Li P, Banjade S, Cheng HC, et al Phase transitions in the assembly of multivalent signalling proteins. Nature. 2012; 483(7389):336‐340. 22398450 10.1038/nature10879 PMC3343696

[8] New LA, Martin CE, Scott RP, et al Nephrin tyrosine phosphorylation is required to stabilize and restore podocyte foot process architecture. J Am Soc Nephrol. 2016; 27(8):2422‐2435. 26802179 10.1681/ASN.2015091048 PMC4978059

[9] Denhez B, Lizotte F, Guimond MO, Jones N, Takano T, Geraldes P. Increased SHP-1 protein expression by high glucose levels reduces nephrin phosphorylation in podocytes. J Biol Chem. 2015; 290(1):350‐358. 25404734 10.1074/jbc.M114.612721 PMC4281738

[10] Aoudjit L, Jiang R, Lee TH, New LA, Jones N, Takano T. Podocyte protein, nephrin, is a substrate of protein tyrosine phosphatase 1B. J Signal Transduct. 2011; 2011:376543. 22013520 10.1155/2011/376543 PMC3195428

[11] Verma R, Venkatareddy M, Kalinowski A, Patel SR, Salant DJ, Garg P. Shp2 associates with and enhances nephrin tyrosine phosphorylation and is necessary for foot process spreading in mouse models of podocyte injury. Mol Cell Biol. 2015; 36(4):596‐614. 26644409 10.1128/MCB.00956-15 PMC4751697

[12] Uchida K, Suzuki K, Iwamoto M, et al Decreased tyrosine phosphorylation of nephrin in rat and human nephrosis. Kidney Int. 2008; 73(8):926‐932. 18256598 10.1038/ki.2008.19

[13] Coward RJM, Welsh GI, Koziell A, et al Nephrin is critical for the action of insulin on human glomerular podocytes. Diabetes. 2007; 56(4):1127‐1135. 17395751 10.2337/db06-0693

[14] Wasik AA, Polianskyte-Prause Z, Dong MQ, et al Septin 7 forms a complex with CD2AP and nephrin and regulates glucose transporter trafficking. Mol Biol Cell. 2012; 23(17):3370‐3379. 22809625 10.1091/mbc.E11-12-1010 PMC3431928

[15] Hartleben B, Schweizer H, Lübben P, et al Neph-Nephrin proteins bind the Par3-Par6-atypical protein kinase C (aPKC) complex to regulate podocyte cell polarity. J Biol Chem. 2008; 283(34):23033‐23038. 18562307 10.1074/jbc.M803143200 PMC2516981

[16] Hussain S, Romio L, Saleem M, et al Nephrin deficiency activates NF-kappaB and promotes glomerular injury. J Am Soc Nephrol. 2009; 20(8):1733‐1743. 19497968 10.1681/ASN.2008111219 PMC2723981

[17] Shih NY, Li J, Cotran R, Mundel P, Miner JH, Shaw AS. CD2AP localizes to the slit diaphragm and binds to nephrin via a novel C-terminal domain. Am J Pathol. 2001; 159(6):2303‐2308. 11733379 10.1016/S0002-9440(10)63080-5 PMC1850607

[18] Schwarz K, Simons M, Reiser J, et al Podocin, a raft-associated component of the glomerular slit diaphragm, interacts with CD2AP and nephrin. J Clin Invest. 2001; 108(11):1621‐1629. 11733557 10.1172/JCI12849 PMC200981

[19] Huber TB, Hartleben B, Kim J, et al Nephrin and CD2AP associate with phosphoinositide 3-OH kinase and stimulate AKT-dependent signaling. Mol Cell Biol. 2003; 23(14):4917‐4928. 12832477 10.1128/MCB.23.14.4917-4928.2003 PMC162232

[20] Fornoni A, Jeon J, Varona Santos J, et al Nephrin is expressed on the surface of insulin vesicles and facilitates glucose-stimulated insulin release. Diabetes. 2010; 59(1):190‐199. 19833886 10.2337/db09-0655 PMC2797921

[21] Kuusniemi AM, Kestilä M, Patrakka J, et al Tissue expression of nephrin in human and pig. Pediatr Res. 2004; 55(5):774‐781. 14764915 10.1203/01.PDR.0000117842.10241.2C

[22] Kapodistria K, Tsilibary EP, Politis P, Moustardas P, Charonis A, Kitsiou P. Nephrin, a transmembrane protein, is involved in pancreatic beta-cell survival signaling. Mol Cell Endocrinol. 2015; 400:112‐128. 25448064 10.1016/j.mce.2014.11.003

[23] Jeon J, Leibiger I, Moede T, et al Dynamin-mediated nephrin phosphorylation regulates glucose-stimulated insulin release in pancreatic beta cells. J Biol Chem. 2012; 287(34):28932‐28942. 22718751 10.1074/jbc.M112.389452 PMC3436561

[24] Villarreal R, Mitrofanova A, Maiguel D, et al Nephrin contributes to insulin secretion and affects mammalian target of rapamycin signaling independently of insulin receptor. J Am Soc Nephrol. 2016; 27(4):1029‐1041. 26400569 10.1681/ASN.2015020210 PMC4814188

[25] Yesil P, Michel M, Chwalek K, et al A new collagenase blend increases the number of islets isolated from mouse pancreas. Islets. 2009; 1(3):185‐190. 21099271 10.4161/isl.1.3.9556

[26] Uhlen M, Fagerberg L, Hallstrom BM, et al Tissue-based map of the human proteome. Science. 2015; 347(6220):1260419. 25613900 10.1126/science.1260419

[27] Karlsson M, Zhang C, Méar L, et al A single–cell type transcriptomics map of human tissues. Sci Adv. 2021; 7(31):eabh2169. 34321199 10.1126/sciadv.abh2169 PMC8318366

[28] Speir ML, Bhaduri A, Markov NS, et al UCSC cell browser: visualize your single-cell data. Bioinformatics. 2021; 37(23):4578‐4580. 34244710 10.1093/bioinformatics/btab503 PMC8652023

[29] Enge M, Arda HE, Mignardi M, et al Single-cell analysis of human pancreas reveals transcriptional signatures of aging and somatic mutation patterns. Cell. 2017; 171(2):321‐330.e14. 28965763 10.1016/j.cell.2017.09.004 PMC6047899

[30] Williamson CR, Jones N. Supplemental File: Reduced nephrin tyrosine phosphorylation enhances insulin secretion and increases glucose tolerance with age. University of Guelph Research Data Repositories. doi:10.5683/SP3/TANJCP. Date of deposit 28 June 2024. 10.1210/endocr/bqae078 PMC11247170 38954536

[31] Casimiro I, Stull ND, Tersey SA, Mirmira RG. Phenotypic sexual dimorphism in response to dietary fat manipulation in C57BL/6J mice. J Diabetes Complications. 2021; 35(2):107795. 33308894 10.1016/j.jdiacomp.2020.107795 PMC7856196

[32] Svensson AM, Östenson CG, Jansson L. Age-induced changes in pancreatic islet blood flow: evidence for an impaired regulation in diabetic GK rats. Am J Physiol Endocrinol Metab. 2000; 279(5):E1139‐E1144. 11052970 10.1152/ajpendo.2000.279.5.E1139

[33] Zhu M, Liu X, Liu W, Lu Y, Cheng J, Chen Y. Β cell aging and age-related diabetes. Aging (Albany NY). 2021; 13(5):7691‐7706. 33686020 10.18632/aging.202593 PMC7993693

[34] Lehtonen S, Lehtonen E, Kudlicka K, Holthöfer H, Farquhar MG. Nephrin forms a complex with adherens junction proteins and CASK in podocytes and in Madin-Darby canine kidney cells expressing nephrin. Am J Pathol. 2004; 165(3):923‐936. 15331416 10.1016/S0002-9440(10)63354-8 PMC1618613

[35] Kim EY, Choi KJ, Dryer SE. Nephrin binds to the COOH terminus of a large-conductance Ca2+-activated K+ channel isoform and regulates its expression on the cell surface. Am J Physiol Renal Physiol. 2008; 295(1):F235‐F246. 18480178 10.1152/ajprenal.00140.2008 PMC2494500

[36] Wasik AA, Dumont V, Tienari J, et al Septin 7 reduces nonmuscle myosin IIA activity in the SNAP23 complex and hinders GLUT4 storage vesicle docking and fusion. Exp Cell Res. 2017; 350(2):336‐348. 28011197 10.1016/j.yexcr.2016.12.010 PMC5243148

[37] Verma R, Kovari I, Soofi A, Nihalani D, Patrie K, Holzman LB. Nephrin ectodomain engagement results in Src kinase activation, nephrin phosphorylation, Nck recruitment, and actin polymerization. J Clin Invest. 2006; 116(5):1346‐1359. 16543952 10.1172/JCI27414 PMC1401486

[38] Blasutig IM, New LA, Thanabalasuriar A, et al Phosphorylated YDXV motifs and Nck SH2/SH3 adaptors act cooperatively to induce actin reorganization. Mol Cell Biol. 2008; 28(6):2035‐2046. 18212058 10.1128/MCB.01770-07 PMC2268406

[39] Thurmond DC, Gonelle-Gispert C, Furukawa M, Halban PA, Pessin JE. Glucose-stimulated insulin secretion is coupled to the interaction of actin with the t-SNARE (target membrane soluble N-ethylmaleimide-sensitive factor attachment protein receptor protein) complex. Mol Endocrinol. 2003; 17(4):732‐742. 12554769 10.1210/me.2002-0333

[40] Hammar E, Tomas A, Bosco D, Halban PA. Role of the Rho-ROCK (Rho-associated kinase) signaling pathway in the regulation of pancreatic beta-cell function. Endocrinology. 2009; 150(5):2072‐2079. 19106222 10.1210/en.2008-1135

[41] Martin CE, New LA, Phippen NJ, et al Multivalent nephrin-Nck interactions define a threshold for clustering and tyrosine-dependent nephrin endocytosis. J Cell Sci. 2020; 133(4):jcs236877. 31974115 10.1242/jcs.236877

[42] Lee EY, Hughes JW. Rediscovering primary cilia in pancreatic islets. Diabetes Metab J. 2023; 47(4):454‐469. 37105527 10.4093/dmj.2022.0442 PMC10404530

[43] Almaça J, Weitz J, Rodriguez-Diaz R, Pereira E, Caicedo A. The pericyte of the pancreatic islet regulates capillary diameter and local blood flow. Cell Metab. 2018; 27(3):630‐644.e4. 29514070 10.1016/j.cmet.2018.02.016 PMC5876933

